# An Anomaly Intrusion Detection for High-Density Internet of Things Wireless Communication Network Based Deep Learning Algorithms

**DOI:** 10.3390/s23010206

**Published:** 2022-12-25

**Authors:** Emad Hmood Salman, Montadar Abas Taher, Yousif I. Hammadi, Omar Abdulkareem Mahmood, Ammar Muthanna, Andrey Koucheryavy

**Affiliations:** 1 Department of Communications Engineering, College of Engineering, University of Diyala, Baquba 32001, Iraq; 2 Department of Medical Instruments Engineering Techniques, Bilad Alrafidain University College, Diyala 32001, Iraq; 3 Department of Telecommunication Networks and Data Transmission, The Bonch-Bruevich Saint-Petersburg State University of Telecommunications, 193232 Saint Petersburg, Russia

**Keywords:** Artificial Neural Network (ANN), Convolutional Neural Network (CNN), Long Short Term Memory (LSTM), IoT, deep learning, intrusion detection system (IDS)

## Abstract

Telecommunication networks are growing exponentially due to their significant role in civilization and industry. As a result of this very significant role, diverse applications have been appeared, which require secured links for data transmission. However, Internet-of-Things (IoT) devices are a substantial field that utilizes the wireless communication infrastructure. However, the IoT, besides the diversity of communications, are more vulnerable to attacks due to the physical distribution in real world. Attackers may prevent the services from running or even forward all of the critical data across the network. That is, an Intrusion Detection System (IDS) has to be integrated into the communication networks. In the literature, there are numerous methodologies to implement the IDSs. In this paper, two distinct models are proposed. In the first model, a custom Convolutional Neural Network (CNN) was constructed and combined with Long Short Term Memory (LSTM) deep network layers. The second model was built about the all fully connected layers (dense layers) to construct an Artificial Neural Network (ANN). Thus, the second model, which is a custom of an ANN layers with various dimensions, is proposed. Results were outstanding a compared to the Logistic Regression algorithm (LR), where an accuracy of 97.01% was obtained in the second model and 96.08% in the first model, compared to the LR algorithm, which showed an accuracy of 92.8%.

## 1. Introduction

The development of telecommunications has been proceeding at a rather rapid rate. The development of new communication technologies has been the driving force behind the rise of many civilizations. Wireless communications have come a long way since the days of smoke signals, pigeons, and hawks, before we finally achieved the level of worldwide connectedness we have today. Alongside facilitating voice and data connectivity, telecommunications have had a profound effect on society by enhancing the quality of life and equipping citizens to deal with calamities and other less severe issues of daily living. The. “Automation of Everything” is the end outcome of contemporary industrialization, which brings about profound change and advances in human civilization. In order to link smart phones and other digital gadgets, it utilizes telecommunication networks. Additionally, it mines data and manages real-world applications. The possibility presented by this transformation enables everyone to gain access to billions of pieces of data and information, which in turn creates exciting challenges. People might notice substantial improvements in quality of life and economic well-being as a consequence of substantial efficiencies gained in both the physical and digital domains. This could lead to a wealthier society. Researchers anticipate that the number of Internet-of-things (IoT) links would therefore attain 83 billion by the year 2024, which itself represents the massive increase in IoT equipment that influences our daily lives across their various types of services in many significant areas, including telemedicine, education, and home automation. This growth will be reflected in the fact that scientists consider the number of IoT interconnections to reach 83 billion by the year 2024 [1]. Since these electronics are connected to the internet and accessible around the clock, it is simple to obtain accurate data in a timely manner [2,3]. As a result, the proliferation of wireless telecommunication infrastructures, wireless handheld devices (such as mobiles), and wireless communication networks has made it possible to organize massive amounts of data at once. Nodes in an Internet-of-Things network typically have low capacity, limited resources, and very few instances of manual control when compared to nodes in conventional networks. As a result, these very unassuming pieces of technology frequently leave themselves open to attack. Concern for the safety of these appliances is on the rise as a result of the daily emergence of new forms of cyberattack. Countless security mechanisms have been created throughout the years, and some of them have proven to be effective at stopping particular types of attacks [4]. Moreover, because the IoT creates such a large volume of data, we need efficient and quick methodologies for detecting attacks. Botnets, denial-of-service (DoS), man-in-the-middle attacks, infiltration, identity theft, data theft, ransomware, etc. are all common forms of assaults in IoT communication channels. Botnet threats are increasingly widespread, and it’s impossible to completely stop them because of how they evolve over time. That’s why there are so many threats and security flaws aimed at these systems.

## 2. Related Work and Literature Review

As a result, aggressive, inexpensive, and reliable wireless intrusion detection systems (IDSs) to prevent and recover from such attacks [5,6] must be developed. Due to the inability of current firewalls to detect and block such a current cybersecurity attack scenario, the secrecy, reliability, and stability of the communication network are at risk of attacks, network attacks, or intrusions that are conveyed over communication network packets. Furthermore, with the widespread use of smart digital devices in an IoT communication network, secure communications among such interconnected devices are a necessity due to the complexity and expense of removing network vulnerabilities from such a system [7].

Despite this, IDS is nevertheless one of the most popular methods. With the help of IDS, communications may be scanned in real time for any signs of malicious behavior or policy violations real time for any signs of malicious behavior or policy violations. Signature-based IDSs and anomaly-based IDSs are the two main groups [4]. Using a method called “signature matching”, signature-based intrusion detection systems can identify potential threats. Signature-based IDS is great at identifying common assaults, but it can also spot less common ones. The potential of anomaly-based IDS to identify previously unseen assaults is impressive. In order to recognize novel and varying forms of natural attacks, this detection approach employs machine/deep learning algorithms. There has been a lot of interest from both academia and industry in using deep learning (DL) algorithms for the design and development of cybersecurity solutions in recent years. The vast amounts of data generated by industrial systems present a huge opportunity for DL techniques to improve upon previous efforts. Nonetheless, there is room for improvement in the development of IoT intrusion investigative techniques [8]. Datasets for the purpose of planning IDSs using machine learning have been developed by a number of laboratories. Researchers working in the field of cybersecurity are paying close attention to the most recent cyberattacks, which are reflected in the UNSW-NB15 dataset [9]. The Cyber Security Research Group at the Australian Centre for Cyber Security (ACCS) has developed a new dataset for testing IDS called UNSW-NB15, which includes over 2 million labels of both normal and aberrant network traffic from the contemporary day [10].

Experiments ranged across the chosen dataset, which was UNSW-NB 15, based on the sort of assault, the protocol that was utilized, or the strategy that was taken to threat identification. As a result, some individuals chose to reduce the complexity of the detecting circuit so that it could identify just one particular incident, or possibly even two different strikes at the very most. Some also started talking about the issue of reliance on the transport layer protocol, such as TCP or UDP. Someone else did not complete the multiclass categorization, but the binary classification was sufficient for them. In recent years, methods from the field of machine learning (ML) have been used to resolve numerous issues in the field of intrusion detection, including the identification of botnets. On the one hand, from the point of view of DL, the problem is appealing due to the huge number of dimensions that it contains. On the contrary, the issue might be remedied with the application of appropriate algorithms for feature selection or dimension minimization. Two of the most well-known IDS datasets that are currently available are the KDD99 [11] and UNSW-NB15 [12] datasets. These datasets have been utilized in a wide variety of research projects [13,14,15,16,17,18,19,20,21,22,23].

One such hybrid approach to categorization is described in [13], whereby the artificial fish swarm (AFS) and the artificial bee colony (ABC) approaches are combined. Both the UNSW-NB15 and the NSL-KDD [24] datasets were used to evaluate the hybrid approach. The wrapper strategy presented in [14] was evaluated on the KDD99 and UNSW-NB15 datasets with several decision-tree classifiers. The C4.5 and enhanced K-means hybrid given in [17] was assessed with the KDD99. An extreme learning machine (ELM) and support vector machine (SVM) hybrid classification strategy was evaluated using the KDD99 in [16,18]. The authors of [19] presented and assessed a K-means/information gain ratio (IGR) hybrid categorization algorithm with the KDD99 dataset. Researchers in [20] presented a method for integrating datasets, which (called MapReduce). They put the new pairing technique through its paces on the KDD99 and DARPA datasets [25]. The merged and purified dataset was then examined with K2 and NaiveBayes (NB) methods.

To test out a novel scaling method for SVMs, researchers in [21] analyzed the UNSW-NB15 dataset. Alternatively, a thorough examination of using the local clustering strategy to address the IDS problem was provided by the authors in [22]. The KDD99 data set was used for this analysis. Contrarily, the multi-layer SVM was examined on the KDD99 dataset in the study presented in [23]. To test how well their suggested methodology worked, they took a few random items from the full dataset. In order to address this issue, the researchers in [15] created a unique discrete metaheuristic algorithm (MHA) called the discrete cuttlefish algorithm (D-CFA). In an effort to streamline the KDD99 dataset, the D-CFA was put to the test. Using the cuttlefish’s unique color-reflection and visibility mechanism, the algorithm was developed. There were a few more iterations of the algorithm that were suggested in the research [26,27]. Nevertheless, in the study reported in [15], a decision tree (DT) classification was used to analyze the features that were picked by the D-CFA. According to the findings of the study, the classifier used only five attributes but attained a detection rate of 91% and a false-positive rate of 3.9% using just those five attributes. In addition, there have been very few attempts by researchers to examine the KDD99 and UNSW-NB15 datasets [10,28,29,30,31,32,33,34]. When analyzing the UNSW-NB15 dataset, the research in [29] utilized a clustering approach in addition to an integrated rule-based IDS. An analysis in a study that is found in [30] looked at the connection between the assaults on the UNSW-NB15 and the transport layer protocols they used.

The authors of [31] provided a prototype that is based on the KDD99 database collection. According to the report, there is a dearth of studies in the field of IDS that examine the existing database. Features from the KDD99 and UNSW-NB15 datasets were studied in [32] to see how effective they are. In their tests, they employed a set of preexisting classifiers alongside an association rule mining technique. According to the study, UNSW-NB15 outperforms KDD99 in terms of detection accuracy and false alarm rate. In light of their findings, the authors of [28] recommend an enhanced version of the KDD99 dataset they call NSL-KDD. Nonetheless, an evaluation of the KDD99 was also provided in [10]. In addition, they looked at the NSL-KDD and KDDcup datasets for further variations. The investigation in [10] was conducted with the intention of improving the datasets by decreasing the number of dimensions, filling in missing values, and getting rid of duplicate instances. According to the results of the research, KDD99 has a large amount of unnecessary repetitions. The correlation between characteristics and KDD99 classes was evaluated using rough-set theory (RST) in [33]. A small subset of features was eliminated from consideration in the analysis since it did not contribute to any of the classes in the dataset. The feature significance of the KDD99 was analyzed by the authors of [34] utilizing an information gain. The research found that some attributes in the collection did not help in the attack uncovering process. The results likewise showed that the challenging set of the database had unique properties compared to the training sample. A most recent research presented in [35], they have used a most recent ML method called Slime Mould Algorithm (SMA). This work integrates SMA into a WSN IDS for anomaly detection. SMAs reduce dataset features from forty-one to five. Although it performed properly, but the algorithm suffers from the early mature problem.

Recent research presented in [36] examined the datasets used in IDS studies and provided an in-depth review of the characteristics of each. During the study, the year and course offerings were considered the most fundamental characteristics. The second feature was the nature of the data, which included the dataset’s structure and any metadata it contained. The third characteristic was the captured packets’ size and duration. The fourth characteristic was the setting in which the recordings were made, which revealed the channels and services utilized by the network to produce the dataset. Finally, the researchers were given an evaluation section, complete with things such as a class-balanced and predetermined data-split. Nonetheless, rather than attempting to encompass all potential attacks, the authors advised researchers to create a dataset that is concentrated on particular threat vectors. The dataset is sufficient if and only if it can be used for the intended purpose. Moreover, in the work given in [36], the exhaustive database was defined to have publicly accessible, accurately categorized categories, to have included real-world network activity rather than synthetic activity, to include all types of assaults, and to be continuously updated. Both the packet header and data payload should be recorded over an extended time period. The UNSW-NB15 was suggested as a generic suggestion for IDS testing due to the large number of assaults it contains compared to the other accessible datasets.

Full KDD99, corrected KDD99, and 10% KDD99 versions, NSL-KDD, UNSW-NB15, center for applied internet data analysis (CAIDA), Australia Defense Force Academy Linux dataset (ADFA-LD), and University of New Mexico (UNM) datasets were all examined in [37]. Consequently, in [38] they provided overviews of all the datasets, with special focus on UNSW-NB15. Accuracy, precision, and recall were reported using the k-nearest neighbors (k-NN) predictor for evaluation purposes across all datasets considered. The classification performance improved using the NSL-KDD, the results showed. According to them, the NSL-KDD’s performance can be attributed to the fact that the dataset has fewer duplicates and is spread more evenly. The study report in [39] investigated the KDD99, with UNSW-NB15, and NSL-KDD databases, utilizing a deep neural network (DNN) for the purposes of IoT networks. Results demonstrate that DNN achieved an accuracy above 90% across the board using the same assessment criteria as in [40] using the F1-measure. DNN also achieved better results than its rivals on the UNSW-NB15 benchmark. The features in both the NSL-KDD and the UNSW-NB15 were evaluated using four-filter-based feature-selection measures: correlation, consistency, information gain, and distance measures. Four classifiers (k-NN, Random Forest (RF), SVM, and deep belief network) were used to evaluate the features selected from the aforementioned methods and disclose the efficiency of the training and testing procedures. With the intention of assisting cybersecurity researchers in their quest for more efficient IDS, this paper reports the features selected for each feature selection method alongside the classification outcomes.

Using a neural network, the researchers in [41] evaluated the UNSW-NB15 dataset to determine which attributes were most relevant. The features are divided into five categories based on their function, including flow, content, time, necessary, and optional. Thirty-one permutations of these characteristics were considered and discussed. Throughout [42], 93% accuracy was achieved using 39 attributes from the different classes. In addition, the research employed a meta predictor called Select-From-Model to choose the combination of 23 attributes based on their scores. When compared to the original set of 39 features, the accuracy achieved by the final set of 23 was significantly higher (97%).

The characteristics in the UNSW-NB15 dataset were compared in [43] to a few feature vectors that have been proposed in literary works. Supervised ML was used to show the processing speeds and accuracy of the classifications. The study’s findings show that existing vectors can be enhanced by making them smaller and by modifying them to handle encrypted communication. In [44], researchers suggested a genetic algorithm, a grey wolf optimizer, particle swarm optimization, and firefly optimization based feature-selection technique. Testing was carried out using the UNSW-NB15 dataset. SVM and J48 classification methods were used to assess the features chosen by the suggested technique. Several feature mixes were tested on the UNSW-NB15 dataset, and their categorization efficacy was revealed. Using the KDD99 dataset, the authors of [45] present and evaluate a hierarchical IDS that employs ML and knowledge-based techniques. In [46], the authors investigated the performance of several ML models, including the RF and gradient-boosting machines, in practical IoT environments. Data-poisoning assaults were simulated by modifying the training data of the datasets using a stochastic algorithm so that the analysis could be carried out. Throughout the study’s tests, researchers used the UNSW-NB15 and ToN_IoT [9] datasets. 

Thus, from the above literature review, we can deduce that there is room for more investigation. That is, different datasets have different attributes and different dimensions. Therefore, it is necessary to introduce a technique that is more practical than the traditional approaches. In other words, there is a need for a technique that has the capacity to deal with various dimensions and different features. Artificial Neural Networks (ANN) are one of the promising approaches that could handle such phenomena. Alongside that, the DNN is another methodology that may also be utilized for this task. However, it will be shown that DNN is not always dominant and that ANN may be more practical. 

Since the UNSW-NB15-dataset focuses primarily on the Internet-of-Things’ communications infrastructure networks, we decided to use it for this particular piece of research. In addition, the dataset was preprocessed, cleaned, and then feature/dimensionality reduction was carried out so that the classification phase could be improved. The ANN-based models and the deep neural network (DNN) models were utilized in the implementation of the classification phase. As a result, the following is a concise summary of the most important contributions made by this work:

- It developed a deep learning-based IDS architecture for anomaly detection by conducting asynchronous security scans on a variety of IoT devices and evaluating the traffic patterns on those devices. The deep learning approach that was proposed can be used to give Internet-of-Things devices the ability to adapt to the dynamic and ad hoc environments in which they operate. After that, the suggested model is subjected to a series of tests to determine whether or not it is accurate and whether or not it is ready for deployment. It offered us outstanding findings which guarantee that the model will be superior to the traditional alternatives that are already in place. We were able to keep our model lightweight while also improving its accuracy, precision, and f-score.
- For the anomaly-based IDS, a second DL model based on a Convolutional Neural Network (CNN) and long-short term memory (LSTM [47]) combination was developed. This second structure was trained and evaluated using various measures.
- The performance of the ANN will be shown to be a technique that is more practical than DNN (CNN plus LSTM combination) for the purpose of IDS when using UNSW-NB15-dataset than others.

Following this structure, the rest of the paper will discuss the following: In Section 3, the proposed models will be shown in detail. Next, in Section 4, we go over the steps taken to get the UNSW-NB15 dataset ready for this research and show the results it the discussion. In Section 5, we sum up our findings.

## 3. Proposed Models

In this paper, two models have been suggested: the first is based on deep learning networks, while the second is a conventional Artificial Neural Network (ANN). That is, the first model for the classification process is a combination of LSTM and CNN layers. LSTM networks are recurrent neural networks. The results of previous computations are used as information feeds in the current iteration of a Recurrent Neural Network (RNN) [48]. They (Hochreiter and Schmidhuber [47]) created the LSTM. The RNN’s inability to predict a word from its long-term memory was addressed, along with the fact that it now provides more precise forecasts based on more current data. RNN’s performance degrades with increasing gap size. By design, LSTM is capable of storing data for a very long time. Time-series data may be processed, predicted, and classified using this method. 

It was stated in [49] that the CNN is a deep learning network design that takes advantage of automatic feature extraction to learn directly from input. When it comes to recognizing objects, persons, and scenes in photos, CNNs shine because of their ability to analyze images for recurring patterns. They may also perform admirably when used to categorize data other than images, such as audio, time series, and signal data. CNNs are essential for application areas that need object detection and computer vision, such as autonomous cars and facial recognition software. There are three main reasons why CNNs are often used for deep learning; by learning the characteristics themselves, CNNs obviate the need for human intervention in the feature extraction process. Using a CNN, one may expect precise recognition performance. In order to expand upon current networks, CNNs may be retrained for other recognition tasks. Tens or even hundreds of layers can be used in a CNN, with each layer learning to identify a unique aspect of the input data. Each piece of training data is sent through a series of filters with varying granularities, with the results feeding into the next layer of the network’s architecture. The filters might begin with really basic features and advance to more complicated attributes that characterize the item in question in a particular way. The second model is an ANN network composed of ten layers: an input layer, an output layer, and eight hidden layers. 

Table 1 shows the structure of the first model, while Table 2 shows the second model. However, the total number of learnable parameters in the first model was 68574, with zero non-trainable parameters. The last layer (No. 13 in Table 1) is the fully connected layer for the classification. As a result, its size is only ten, because it will produce ten classes. Note that the first layer of the first model is not shown in Table 1. The first layer after the input layer is a 1-dimensional (1D) convolutional layer (No. 1 in Table 1). Then activation layer using Rectified Linear Unit (LeLU). The ReLU function, short for Rectified Linear Unit, is a piecewise linear function that returns the input value unmodified if it is positive and 0 otherwise. After that, a thirty-two-filter convolutional layer of 1D, ReLU-activation. This was the first group in the structure. The next group is starting a convolution layer of kernel size 64, ReLU-activation, dropout layer of ratio 0.2, a convolutional layer, ReLU-activation, and a dropout layer. Up to this point, the total number of learnable parameters are 42,944 trainable parameters. Last but not least, the third group consists of an LSTM layer of size 40, which has 16,800 trainable parameters, followed by another LSTM layer of size 30, which involves 8520 trainable parameters, and finally a fully connected layer of size 10, which has 310 trainable parameters, as listed in Table 1. 

The second model was constructed, as in Table 2, from nine fully connected layers (dense layer) corresponding to sizes (1, 300), (1, 400), (1, 600), (1, 800), (1, 1), (1, 500), (1, 400), (1, 1), and (1, 10) which is the output layer, respectively. The total trainable parameters in the second model, the ANN-model, are 1,061,522 parameters, with zero non-trainable parameters, as shown in Table 2. The sequence of the layers, both in Table 1 and Table 2, stands for the flow of the data from the input to the output. Thus, these two tables can be imagined as model structures.

In this effort, we create an IDS with DL algorithms powering its backend. The result is a system that can adapt to changing needs and grow with the business. The success of any machine learning or deep learning algorithm is determined by the caliber of the data used in the algorithm. On the other hand, the model’s accuracy increases as more data is added to its training set. Thus, it is crucial in these IDSs to ensure the quality of the dataset in order to identify and mitigate Bot-Net assaults on IoT sensors. In this research, we employ the most recent dataset available (the UNSW-NB15 dataset) and conduct additional evaluations of the model with a variety of assessment techniques. However, there are a total of 2,540,044 entries in the collection, which includes nine different types of assaults (Fuzzers, Analysis, Backdoors, DoS, Exploits, Generic, Reconnaissance, Shellcode, and Worms). A subset of this data was split up and used as a training set and a test set. There are a total of 175,341 records of both attack and normal types in the training set, while there are 82,332 records in the testing set. The six types of features, which contribute to the dataset are the Additional Generated Features (AGF), Time Features (TF), Content Features (CF), Basic Features (BF), Class Features, and Flow Features (FF). The AGF can also be broken down into its two constituent parts: Connections and General Purpose Features.

## 4. Simulation Results and Discussion

The investigations in this paper were run on a Windows-10 64-bit computer using the Python libraries Scikit-Learn and Keras-Tensorflow. These ML/DL libraries are widely used in machine learning, deep learning, and data science. With regards to hardware, we ran our simulations on a 1.60GHz 2.30GHz Intel Core i5-4200U CPU with 16GB of RAM. However, Table 3 lists all of the UNSW-NB15 dataset-features. Note that the Id is discarded from the list shown in Table 3. The first process to be achieved is to drop the feature No. 43, F43, and the last feature, F44, which will be used later. There are eleven features of type float64, four features of type object, and thirty features of type int64, including the Id, which is discarded from Table 3. 

Consequently, to prepare the dataset, Id is the first function to be removed. This is only a search field and not a descriptive one, as indicated at the beginning of this section and in Table 3. After “attack cat,” this is the next functionality to be removed. Since this feature is a superset of the target feature, it will yield perfect predictions but not generalizability. However, for some distributions, it may be helpful to remove the outliers in order to minimize the skew. The approach employed here cuts features to the 95th percentile if their greatest value is greater than 10 times the median value. If the 95th percentile is really high, we may safely assume that there is more valuable information in the tail than in the central region. Features with bounds more than 10 times the median is the only one subject to the clamping. This keeps us from having to carry out too much pruning, which protects things such as bi-modals and tiny value distributions. According to the statistics of the dataset, which are found in [50], the entries are skewed to the right. Thus, applying the Log-function to the vast majority of numbers since they are slanted to the right.

Since manually applying the log function to each continuous feature would have been a monumental task, a simple rule has been established: if the number of unique entries in the continuous attribute is greater than 50, subsequently implement the log function. To exclude the integer-based characteristics that behave more categorically, it is preferable to find more than 50 distinct values. The cardinality of some characteristics is quite high, but that number is brought down to about five or six in this step. The rule of thumb is to use the five most frequent labels from the attribute as the actual labels and assign the other labels the status of “rarely used.” Additional encoding will not result in an explosion of dimensionality or the constraint of dimensions.

Feature selection [18] is all about selecting features from a large pool of candidates in order to improve accuracy, save training time, and eliminate overfitting selection [26]. There are three distinct varieties. Embedded methods, filter methods, and wrapper methods. While the proper predictor is used to assign a value to a subset of features in wrapper techniques, relevance is determined by correlation with the relying variable in filter methodologies. Since filter techniques do not necessitate training the models, they are quicker and require less computing effort than wrapper approaches. SelectPercentile is a feature selection technique that may be achieved with Scikit-learn [51]. This approach gives each feature a percentile depending on its score, and it is a feature selection method. After that, features might be chosen up to a certain cutoff percentile by taking into consideration a classifier’s overall performance. In this work, the best 80th percentiles have been selected. 

Accordingly, only 37 features have been selected out of 43 features, as listed in Table 4. That is, the features are listed descending manner, from highest score to lowest score. The highest scored features that are in millions are, F16, F19, F6, F9, F17, F18, F7, F30, and F31 corresponding to Sinpkt, Djit, Dpkts, Rate, Dinpkt, Sjit, Sbytes, response_body_len, and ct_srv_src, respectively, where the scores were, respectively, (1719.167, 1422.332, 1191.638, 122.0820, 118.2373, 117.9835, 113.8066, 110.4150, and 105.2134) × 10^3^. Hence, there are nine features with scores in millions, while there are twenty features with scores in hundred-thousands, and the rest are scored less than ten-thousands, as listed in Table 4.

**Figure 1:**
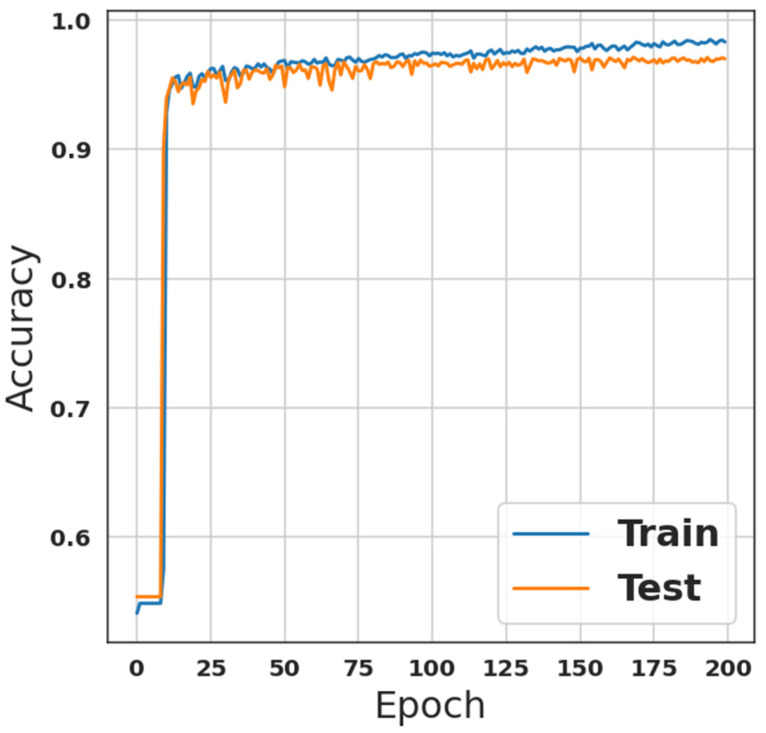
Accuracy of the first model, as shown in Table 1, results.

**Figure 2:**
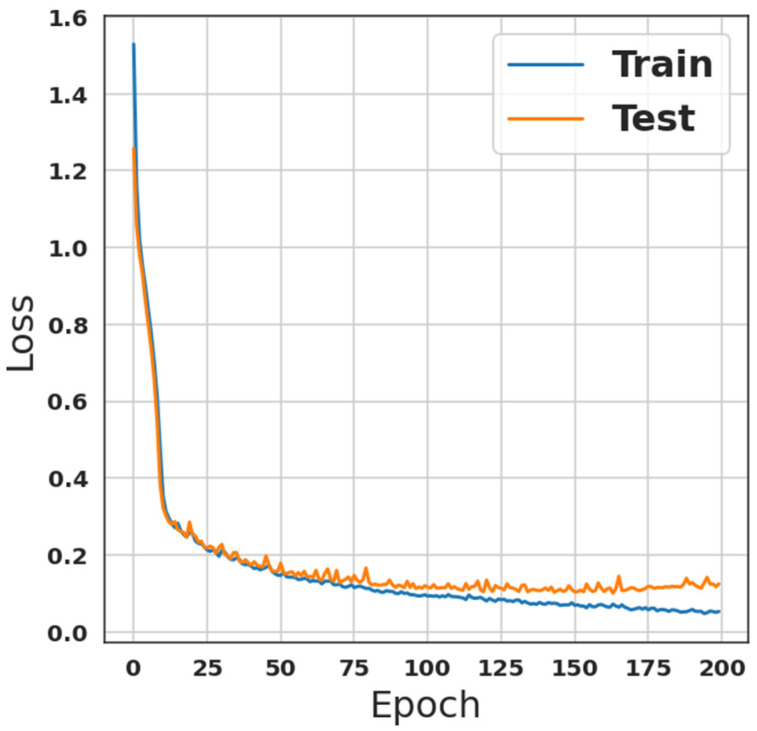
Loss results of the first model which is shown in Table 1.

The final step in preprocessing the dataset is the encoding operation. However, it is necessary to encode the categorical attributes in order to guarantee that the classifiers can understand them. Considering that neither of the categorical characteristics are ordinal, one-hot encoding is utilized in this situation. Consequently, the dataset is ready for the classification algorithms, the last step in the IDS. These algorithms are discussed in the previous section. The training sub-dataset has been fed to the first model see Table 1, which is a custom CNN network combined with a custom LSTM network. The number of iterations/Epochs was two hundred with a batch size of 2000, while the validation ratio was 33%. The model was trained using these parameters, and the results of the training phase, in terms of accuracy and loss, are shown in Figure 1 and Figure 2, respectively. From Figure 1, it is observed that the accuracy was 96.08%, with recall, precision, and F1-score equal to 96.08% each. However, the evaluation loss was 0.0968 while the evaluation accuracy was 96.2%.

**Figure 3:**
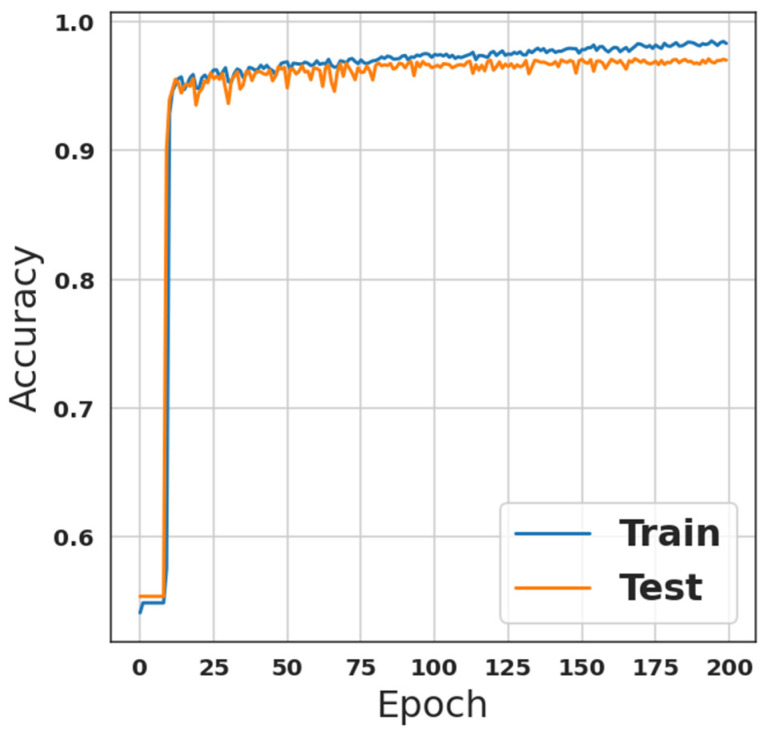
ANN-model accuracy during training-phase.

**Figure 4:**
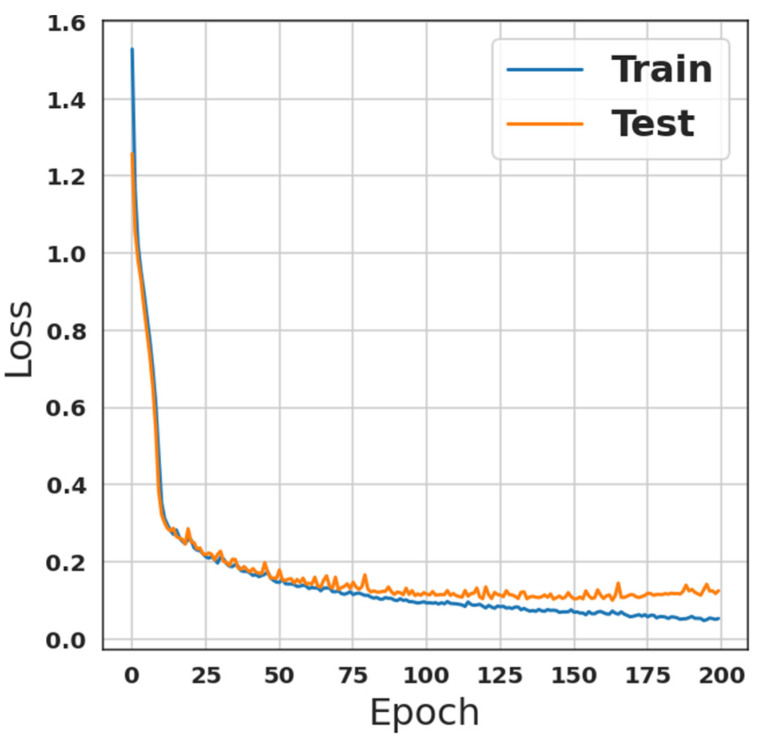
ANN-model loss during training-phase.

Moreover, the second model, which is presented in Table 2, was trained using the same settings as the first model for a fair comparison. This model is an ANN combination of multi-dense layers. As indicated previously in the last section, there are 1,061,522 parameters that are all trainable. Nevertheless, Figure 3 shows the training accuracy of this model, while Figure 4 depicts the loss of the training phase. Although training and prediction time were longer than with the first model, accuracy was improved to 97.01% with recall, precision, and F1-score equal to 97.01% for each of them.

For more convenience, these two models have been compared with the logistic regression algorithm. Using input variables, logistic regression (LR) estimates the likelihood of a discrete result. LR is widely used to model a variable with a true/false or other binary result. For modeling purposes where there are more than two distinct discrete events, multinomial LR is the method of choice. LR is a helpful analysis tool for classification problems, such as deciding which category is the best fit for a new sample. LR is a helpful analytic tool for cyber security because many areas of the field have classification problems, such as threat detection. Despite the low processing time required by the LR algorithm, its accuracy was 92.80%. However, the recall measure achieved by LR was 92.80%. The precision and F1-score are 92.83% and 92.8%, respectively. Table 5 shows a comparison between our two suggested models and the LR algorithm.

That is, the first suggested model, which is represented by the custom CNN + LSTM model in Table 5, outperforms the LR in terms of accuracy, recall, precision, and F1-score measures. Improvements of 3.28%, 3.28%, 3.25%, and 3.28% in the accuracy, recall, precision, and F1-score, respectively. While the second model was superior as compared with both the LR and the first model. Thus, the ANN model improved the accuracy by 0.93% and 4.21% when compared with the first model and the LR approach, respectively. The same improvement amounts were captured for the recall and F1-score, while in terms of precision, the improvement was 0.93% and 4.18% compared to the first model and LR methodologies, respectively. 

## 5. Conclusions

For securing the communication links in IoT networks and other wireless communication networks, two Intrusion Detection System models were built based on deep learning approaches. The first model was an architecture of custom Convolutional Neural Networks combined with Long Short Term Memory layers. The second model was constructed using different dense layer sizes. The two models were trained using a well-known dataset called UNSW-NB15. The dataset was first cleaned, i.e., preprocessed, before it was fed to our two different classification models. Outstanding results were obtained using the two suggested models, as compared to the Logistic Regression algorithm. However, the second model performed better than the first model by less than 1%. Thus, it is proved that the ANN’s performance was superior to that of the custom CNN and LSTM combination. However, a drawback can be noticed, which is the training on a single dataset. If there is more than one dataset, the ANN model may fail. That is, a future work is proposed to enable the ANN to overcome this issue. This can be achieved by combining more than two datasets, or it can be achieved by fine-tuning the pre-trained ANN network to be used with other datasets.

## Figures and Tables

**Table 1:** Deep learning first model layers’ architecture.

Sequence	Layer Type	Output Shape	No. Learnable Parameters
1	Convolution (1D)	(1, 32)	8992
2	Activation (ReLU)	(1, 32)	0
3	Convolution (1D)	(1, 32)	3104
4	Activation (ReLU)	(1, 32)	0
5	Convolution (1D)	(1, 64)	10,304
6	Activation (ReLU)	(1, 64)	0
7	Dropout (0.2)	(1, 64)	0
8	Convolution (1D)	(1, 64)	20,544
9	Activation (ReLU)	(1, 64)	0
10	Dropout (0.2)	(1, 64)	0
11	LSTM	(1, 40)	16,800
12	LSTM	(1, 30)	8520
13	Fully Connected (Dense) (output)	(1, 10)	310

**Table 2:** ANN, second model, layers architecture.

Sequence	Layer Type	Output Shape	No. Learnable Parameters
1	Fully Connected (Dense)	(1, 300)	17,100
2	Fully Connected (Dense)	(1, 400)	120,400
3	Fully Connected (Dense)	(1, 600)	240,600
4	Fully Connected (Dense)	(1, 800)	480,800
5	Fully Connected (Dense)	(1, 1)	801
6	Fully Connected (Dense)	(1, 500)	1000
7	Fully Connected (Dense)	(1, 400)	200,400
8	Fully Connected (Dense)	(1, 1)	401
9	Fully Connected (Dense)	(1, 10)	20

**Table 3:** UNSW-NB15 dataset 42 features.

Attribute	Term	Type	Attribute	Term	Type	Attribute	Term	Type
F1	Dur	Float-64	F16	Sinpkt	Float-64	F31	ct_srv_src	Int-64
F2	Proto	Object	F17	Dinpkt	Float-64	F32	ct_state_ttl	Int-64
F3	Service	Object	F18	Sjit	Float-64	F33	ct_dst_ltm	Int-64
F4	State	Object	F19	Djit	Float-64	F34	ct_src_dport_ltm	Int-64
F5	Spkts	Int-64	F20	Swin	Int-64	F35	ct_dst_sport_ltm	Int-64
F6	Dpkts	Int-64	F21	Stcpb	Int-64	F36	ct_dst_src_ltm	Int-64
F7	Sbytes	Int-64	F22	Dtcpb	Int-64	F37	is_ftp_login	Int-64
F8	Dbytes	Int-64	F23	Dwin	Int-64	F38	ct_ftp_cmd	Int-64
F9	Rate	Float-64	F24	Tcprtt	Float-64	F39	ct_flw_http_mthd	Int-64
F10	Sttl	Int-64	F25	Synack	Float-64	F40	ct_src_ltm	Int-64
F11	Dttl	Int-64	F26	Ackdat	Float-64	F41	ct_srv_dst	Int-64
F12	Sload	Float-64	F27	Smean	Int-64	F42	is_sm_ips_ports	Int-64
F13	Dload	Float-64	F28	Dmean	Int-64	F43	attack_cat	Object
F14	Sloss	Int-64	F29	trans_depth	Int-64	F44	Label	Int-64
F15	Dloss	Int-64	F30	response_body_len	Int-64			

**Table 4:** List of selected features after preprocessing operation.

Feature	Score (×10^3^)	Feature	Score (×10^3^)	Feature	Score (×10^3^)
F16	1719.167	F11	52.38466	F26	5.255736
F19	1422.332	F36	51.71041	F13	5.078488
F6	1191.638	F29	48.28637	F3	3.278490
F9	122.0820	F24	42.79038	F20	0.3157251
F17	118.2373	F10	34.03161	F21	0.255003
F18	117.9835	F14	24.79605	F35	0. 2291240
F7	113.8066	F5	24.78945	F25	0.2284359
F30	110.4150	F2	20.64451	F22	0.1100448
F31	105.2134	F8	17.47411	F23	0.09680493
F32	99.98646	F15	11.57659	F34	0.02468076
F37	82.63284	F12	9.971425	F33	0.02169648
F27	78.87113	F1	7.812781		
F4	61.40787	F28	6.947356		

**Table 5:** Comparison results of LR, first model (custom CNN + LSTM), and ANN model.

Approach	Accuracy	Recall	Precision	F1-Score
LR	92.8%	92.8%	92.83%	92.8%
Custom CNN + LSTM model	96.08%	96.08%	96.08%	96.08%
ANN model	97.01%	97.01%	97.01%	97.01%
